# Uncertainties in the Relationship between Tibia Lead and Cumulative Blood Lead Index

**DOI:** 10.1289/ehp.10778

**Published:** 2008-03

**Authors:** Norm Healey, David R. Chettle, Fiona E. McNeill, David E. B. Fleming

**Affiliations:** Health Canada, Sidney, British Columbia, Canada, E-mail: norm_healey@hc-sc.gc.ca; McMaster University, Hamilton, Ontario, Canada; Mount Allison University, Sackville, New Brunswick, Canada

Uncertainties in the relationship between bone Pb and cumulative blood lead index (CBLI), including evidence of nonlinearity and differences between the sexes, should be appropriately recognized when setting workplace blood Pb limits to achieve target bone Pb concentrations.

Schwartz and Hu (2007) recommended a maximum occupational tibia Pb concentration of 15 μg/g. They stated that, based on the slope of the relationship between tibia Pb and CBLI calculated by Hu et al. (2007), a tibia Pb of 15 μg/g can be avoided by limiting the CBLI to < 200–400 μg-years/dL.

Hu et al. (2007) acknowledged the uncertainty in the slope of the relationship between tibia Pb and CBLI. However, over the range of cumulative Pb exposures that would produce a tibia Pb concentration of 15 μg/g, the slope of the relationship between tibia Pb and CBLI may be less than the slope of 0.05 [95% confidence interval (CI), 0.046–0.055] μg/g per μg-years/dL calculated by Hu et al. (2007).

Table 1 presents slopes and mean tibia Pb concentrations among subjects of eight published studies. Gerhardsson et al. (1993) reported a slope of 0.022 μg/g per μg-years/dL (no uncertainty reported) and Armstrong et al. (1992) reported a slope of 0.10 (± 0.02) μg/g per μg-years/dL. These represent a greater range of slopes than reported by Hu et al. (2007).

These data also suggest that the tibia Pb versus CBLI slope may not be constant, with lower slopes evident for lower tibia Pb and CBLI levels. This trend has been noted previously (Chettle 2005; Fleming et al. 1997). For tibia Pb concentrations of approximately 15 μg/g, a slope of approximately 0.025 μg/g per μg-years/dL seems equally plausible as the slope calculated by Hu et al. (2007).

A slope of 0.025 μg/g per μg-years/dL yields an allowable CBLI of 600 μg-years/dL, or an average annual blood Pb concentration of 15 μg/dL for 40 working years. This compares to 5–10 μg/dL for 40 working years associated with Schwartz and Hu’s (2007) recommended CBLI of 200–400 μg-years/dL.

These slopes are also based on studies of predominantly male subjects and may not account for differences in Pb toxicokinetics between the sexes (McNeill et al. 2000; Popovic et al. 2005).

## Figures and Tables

**Table 1:** Various slopes of the relationship between tibia Pb and CBLI, and related mean tibia Pb concentration among study subjects.

Study	No.	*r*	Slope^a^	Mean Pb (μg/g tibia bone mineral)
Gerhardsson et al. (1993)	100	0.60	0.022	16.9
Erkkilä et al. (1992)	91	0.66	0.028 ± 0.003	21.1
Somervaille et al. (1988)	79	0.86	0.050 ± 0.003	31.0
Somervaille et al. (1988)	88	0.82	0.060 ± 0.005	32.3
Cake (1994)	53	0.70	0.059 ± 0.009	39
Fleming et al. (1997)	367	0.83	0.056 ± 0.002	40.6
Hu et al. (1991)	12	0.92	0.061 ± 0.008	46
Armstrong et al. (1992)	15	0.87	0.10 ± 0.02	54.8

a Units are μg/g bone mineral per μg-year/dL.

## References

[b1-ehp0116-a0109a] Armstrong R, Chettle DR, Scott MC, Somervaille LJ, Pendlington M (1992). Repeated measurements of tibia lead concentrations by in vivo X ray fluorescence in occupational exposure. Br J Ind Med.

[b2-ehp0116-a0109a] Cake KM (1994). In Vivo X-Ray Fluorescence of Bone Lead in the Study of Human Lead Metabolism [Master’s Thesis].

[b3-ehp0116-a0109a] Chettle DR (2005). Three decades of in vivo x-ray fluorescence of lead in bone. X-Ray Spectrometry.

[b4-ehp0116-a0109a] Erkkilä J, Armstrong R, Riihimaki V, Chettle DR, Paakkari A, Scott M (1992). In vivo measurements of lead in bone at four anatomical sites: long term occupational and consequent endogenous exposure. Br J Ind Med.

[b5-ehp0116-a0109a] Fleming DEB, Boulay D, Richard NS, Robin JP, Gordon CL, Webber CE (1997). Accumulated body burden and endogenous release of lead in employees of a lead smelter. Environ Health Perspect.

[b6-ehp0116-a0109a] Gerhardsson L, Attewell R, Chettle DR, Englyst V, Lundstrom NG, Nordberg GF (1993). In vivo measurements of lead in bone in long-term exposed lead smelter workers. Arch Environ Health.

[b7-ehp0116-a0109a] Hu H, Pepper L, Goldman R (1991). Effect of repeated occupational exposure to lead, cessation of exposure, and chelation on levels of lead in bone. Am J Ind Med.

[b8-ehp0116-a0109a] Hu H, Shih R, Rothenberg S, Schwartz BS (2007). The epidemiology of lead toxicity in adults: measuring dose and consideration of other methodologic issues. Environ Health Perspect.

[b9-ehp0116-a0109a] McNeill FE, Stokes L, Brito JA, Chettle DR, Kaye WE (2000). ^109^Cd K X ray fluorescence measurements of tibial lead content in young adults exposed to lead in early childhood. Occup Environ Med.

[b10-ehp0116-a0109a] Popovic M, McNeill FE, Chettle DR, Webber CE, Lee CV, Kaye WE (2005). Impact of occupational exposure on lead levels in women. Environ Health Perspect.

[b11-ehp0116-a0109a] Schwartz BS, Hu H (2007). Adult lead exposure: time for change. Environ Health Perspect.

[b12-ehp0116-a0109a] Somervaille LJ, Chettle DR, Scott MC, Tennant DR, McKiernan MJ, Skilbeck A (1988). In vivo tibia lead measurements as an index of cumulative exposure in occupationally exposed subjects. Br J Ind Med.

